# Engineering intracellular biomineralization and biosensing by a magnetic protein

**DOI:** 10.1038/ncomms9721

**Published:** 2015-11-02

**Authors:** Yuri Matsumoto, Ritchie Chen, Polina Anikeeva, Alan Jasanoff

**Affiliations:** 1 Department of Biological Engineering, Massachusetts Institute of Technology, 77 Massachusetts Avenue Cambridge, Massachusetts 02139, USA; 2 Department of Materials Science & Engineering, Massachusetts Institute of Technology, 77 Massachusetts Avenue Cambridge, Massachusetts 02139, USA; 3 Research Laboratory of Electronics, Massachusetts Institute of Technology, 77 Massachusetts Avenue Cambridge, Massachusetts 02139, USA; 4 Department of Brain & Cognitive Sciences, Massachusetts Institute of Technology, 77 Massachusetts Avenue Cambridge, Massachusetts 02139, USA; 5 Department of Nuclear Science & Engineering, Massachusetts Institute of Technology, 77 Massachusetts Avenue Cambridge, Massachusetts 02139, USA

## Abstract

Remote measurement and manipulation of biological systems can be achieved using magnetic techniques, but a missing link is the availability of highly magnetic handles on cellular or molecular function. Here we address this need by using high-throughput genetic screening in yeast to select variants of the iron storage ferritin (Ft) that display enhanced iron accumulation under physiological conditions. Expression of Ft mutants selected from a library of 10^7^ variants induces threefold greater cellular iron loading than mammalian heavy chain Ft, over fivefold higher contrast in magnetic resonance imaging, and robust retention on magnetic separation columns. Mechanistic studies of mutant Ft proteins indicate that improved magnetism arises in part from increased iron oxide nucleation efficiency. Molecular-level iron loading in engineered Ft enables detection of individual particles inside cells and facilitates creation of Ft-based intracellular magnetic devices. We demonstrate construction of a magnetic sensor actuated by gene expression in yeast.

## Abstract (web summary)

Magnetic manipulation of biological systems requires the development of improved molecular handles. Here the authors isolate ferritin mutants with enhanced biomineralization from a yeast genetic screen and show their application to cell separation, multiscale imaging, and construction of sensors.

## Graphical abstract

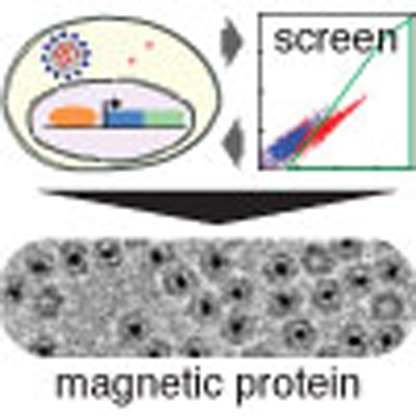

Magnetic approaches to biological experimentation are particularly attractive because they interact minimally with biological processes, rarely incur damage and have already led to powerful manipulation and imaging techniques. Existing magnetic biotechnologies are of limited value for studying molecular and cellular level phenomena, however. The best known magnetic measurement techniques, nuclear magnetic resonance and magnetic resonance imaging (MRI), are ill-suited for analysis of specific molecular phenomena in cells and tissue. *In vivo* nuclear magnetic resonance spectroscopy is too insensitive to permit robust measurements of most biomolecules^1^. Molecular MRI measurements can be made using contrast agents that combine magnetic properties with other functionalities^2^,^3^,^4^,^5^, but these agents need to be delivered exogenously. Techniques for magnetic modulation of biological systems have been demonstrated at cellular level^6^,^7^,^8^, but also tend to depend on exogenous nanoparticles that are difficult to apply to biological systems. Although manipulation of cellular magnetism and magnetic image signals has also been demonstrated using genetic techniques^9^,^10^,^11^,^12^,^13^,^14^, the effects tend to be weaker or less specific than approaches based on synthetic magnetic nanoparticles, in part because the molecular organization of magnetic material in cells is less controlled.

A strongly magnetic protein could provide a basis for robust modulation or detection of well-defined molecular-level phenomena. A promising starting point for generation of such a molecule is ferritin (Ft), an iron storage protein found in most animal, plant and bacterial cells^15^. Ft proteins consist of a spherical shell of 24 identical or closely homologous polypeptide chains, in which a reservoir of hydrated iron oxide accumulates and can be rapidly mobilized according to physiological needs. Ft variants have been used as magnetic gene reporters^12^,^13^, expressible cellular imaging agents^16^,^17^,^18^ and components of magnetically responsive genetic devices^19^, but Ft is much less potent than synthetic nanoparticles of similar volume and often contains far fewer iron atoms than its core structure could in principle accommodate^20^. *In vitro* manipulation of Ft mineralization has enabled the generation of highly magnetic species^21^, but the resulting protein complexes cannot be applied in conjunction with genetic techniques and suffer similar limitations to those of synthetic nanoparticles.

To address these limitations, we designed a strategy for enhancing the magnetic properties of intracellularly expressed Ft in a systematic and high-throughput fashion. In this paper, we present our approach and its success in isolating mutant Ft variants that biomineralize iron more effectively than their natural counterparts. We characterize the selected mutants and show that their enhanced iron loading capability may arise from improvements to iron oxide nucleation. Finally, we show that the new ‘hypermagnetic' Ft variants act as genetically encodable tools for multiscale cellular imaging, magnetic manipulation of cells and construction of intracellular magnetic devices capable of sensing molecular-level phenomena.

## Results

### Screening for Ft variants with enhanced biomineralization

**Figure 1:**
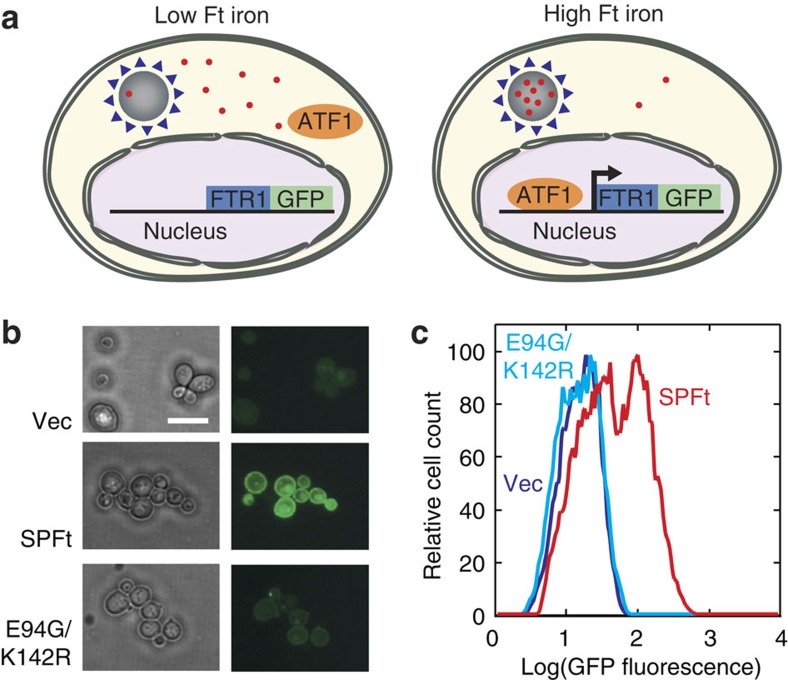
Fluorescent reporter system used to probe intracellular iron mineralization by Ft. (**a**) Schematic diagram of yeast cells containing an iron-responsive reporting system. Sequestration of cytosolic iron (red dots) into Ft (grey) triggers translocation of an iron-responsive transcription factor, AFT1 (orange), into the nucleus, where it induces transcription of an FTR1–GFP fusion protein (blue/green). Iron accumulation by effective Ft variants therefore results in a green signal (right). (**b**) Yeast cells transformed with empty vector (Vec), SPFt and the SPFt mutant E94G/K142R, which lacks iron mineralization capability, were incubated in minimum media overnight. Differences in FTR1–GFP expression are visible in the fluorescence micrographs at right, with SPFt but not E94G/K142R effective at upregulating the reporter; corresponding phase contrast images are shown at left. Scale bar, ∼10 μm. (**c**) FACS histograms showing the distribution of GFP-associated fluorescence observed in yeast cell populations transformed with vector, SPFt and E94G/K142R.

Our biomaterial engineering approach was based on the hypothesis that mutant Ft molecules that sequester iron compounds most effectively would also form complexes with optimal magnetic properties—a view motivated by the fact that both greater Ft iron content^22^ and denser, unhydrated iron oxide mineralization^21^ can result in higher per-particle magnetic moments. Iron accumulation by Ft variants is expected to reduce cytosolic iron concentration by mass action principles, so we established a reporting system in yeast whereby expression of Ft mutants could be evaluated for induction of a low cytosolic iron phenotype. In *Saccharomyces cerevisiae*, intracellular iron level is regulated by the iron-responsive transcriptional activator Aft1, which under low-iron conditions translocates into the nucleus and regulates genes involved in iron uptake^23^. One of the genes upregulated by Aft1 encodes the cell surface high-affinity iron transporter, FTR1^24^; by monitoring expression of an FTR1–green fluorescent protein (GFP) fusion reporter^25^, we could therefore identify individual cells that display low cytosolic iron concentrations (Fig. 1a). This system was intended as a tool for selecting mutant Ft variants that robustly sequester cellular iron, and that would therefore induce greater FTR1–GFP expression and fluorescence than Ft variants with less potent iron binding capacity.

As a template for random mutagenesis and screening, we choose to work with a Ft from the thermophilic bacterium *Pyrococcus furiosus* (PFt). PFt has the advantage that it is highly thermostable (*T*_*m*_>120 °C)^26^, and, therefore, likely to be more tolerant to mutations introduced to alter biomineralization than human heavy chain Ft (HFt; *T*_*m*_=∼77 °C)^27^, which has been used for the majority of biotechnological applications of Ft in the past. In addition, PFt forms homooligomeric protein shells that require only a single polypeptide, in contrast to conventional mammalian Fts that incorporate two chains, making PFt structure and chemistry simpler and more predictable. To facilitate isolation and analysis of PFt variants, we fused an affinity tag (Strep-tag II) to the N terminus of PFt to form a construct abbreviated SPFt (Supplementary Fig. 1a). The tag had minimal effect on protein folding and iron loading functions of the protein (Supplementary Figs 1b–e). SPFt was expressed in yeast cells bearing the FTR1–GFP reporter and induced elevated fluorescence, compared with control cells bearing no SPFt or harbouring a compromised SPFt with E94G and K142R substitutions that eliminate ferroxidase activity of the protein (Fig. 1b). Results of fluorescence microscopy were further validated by fluorescence-activated cell sorting (FACS) analysis (Fig. 1c). Fluorescence histograms from cells transfected with a SPFt expression plasmid displayed a peak of notably higher fluorescence, indicating upregulation of the fluorescent reporter; a second peak with fluorescence comparable to vector control transfected cells was observed under saturating growth conditions and probably arises from SPFt expression plasmid loss in some cells. These results were consistent with the explanation that SPFt expression sequesters cytosolic iron and boosts FTR1–GFP reporter expression.

**Figure 2:**
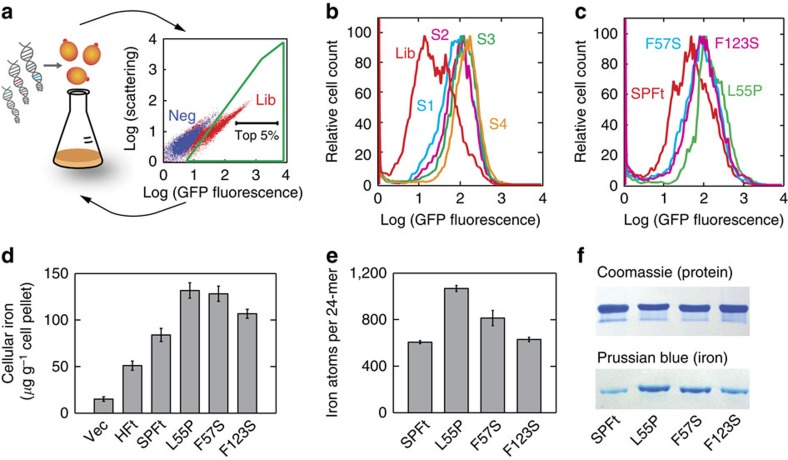
Selection of SPFt mutants by high-throughput genetic screening. (**a**) Summary of the fluorescence-activated cell sorting (FACS)-based yeast genetic screening procedure. Control yeast cells lacking the FTR1–GFP reporter (neg) or positive cells harbouring the reporter and a SPFt gene library (Lib) were grown in minimum media. The yeast populations were presorted to remove debris and aggregated cells, and then used to establish a criterion (green outline) designed to reject cells lacking a functional reporter construct. From among Lib cells that passed this criterion, roughly 5% of cells which displayed the highest GFP fluorescence intensities (black label) were selected during each FACS run. Multiple rounds of selection and regrowth were performed (arrows) to enrich library mutants which induced the highest levels of fluorescent reporter expression. (**b**) A histogram showing the distribution of GFP fluorescence intensity in the yeast cell population transformed with the initial library (Lib, red), and following one to four successive rounds of enrichment (S1–S4). (**c**) Flow cytometry distributions of GFP fluorescence intensity of yeast cells transformed with SPFt (red) and three mutants identified through the screen, L55P (green), F57S (cyan) and F123S (magenta) incubated in minimal media overnight. Cytosolic iron content of intact yeast (**d**) and molecular-level iron loading by purified SPFt variants (**e**) was measured for each of the selected mutants using a bathophenanthrolinedisulfonate-binding assay following 16 h incubation of the corresponding cells in iron-rich medium. Error bars denote s.e.m. of three or more independent measurements. (**f**) Native gel analysis of purified SPFt and mutant nanoparticles stained with Coomassie blue for protein content (top) and Prussian blue for iron content (bottom), showing substantially increased iron content of the selected SPFt mutants.

To isolate mutants that preferentially biomineralize more iron *in vivo*, we subjected the entire PFt coding sequence in SPFt to PCR-based random mutagenesis. After transfection, this resulted in a library of 10 million yeast clones expressing randomly mutated SPFt variants with an average mutation rate of one nucleotide change per gene (Supplementary Fig. 2). This relatively low mutation rate was chosen to avoid accumulation of deleterious mutations, which could obscure beneficial but rare mutations. The yeast library was incubated in a minimum media and sorted by FACS to obtain cells exhibiting highest levels of FTR1–GFP fluorescence. Cells in the top 5% were propagated for a subsequent round of sorting (Fig. 2a), and the procedure was repeated. After four rounds (Fig. 2b), we sequenced the sorted population and identified mutations that were enriched among the selected yeast cells (Supplementary Table 1). Following retesting of the individual-enriched mutations, three were chosen for further analysis: L55P, F57S and F123S.

### Characterization of selected SPFt mutants

To confirm the Ft dependence of iron reporter expression in the selected clones, plasmids for SPFt L55P, F57S and F123S were isolated and retransformed for reanalysis by FACS; fluorescence histograms were consistent with the screening results (Fig. 2c). As an additional test of the iron accumulation phenotype, we incubated the three selected clones in iron-supplemented media and measured the total cellular iron content (Fig. 2d) and iron content of purified SPFt proteins (Fig. 2e,f). The most effective of the SPFt mutants, L55P, induced 1.6±0.2 (mean±s.e., *n*=3) times greater cellular iron accumulation than wild-type SPFt and 2.6±0.3 times greater accumulation than HFt. Compared with SPFt, the L55P mutant also exhibited almost double the number of iron atoms per Ft 24-mer, indicating that the cellular biomineralization phenotype originates largely from an increase in iron sequestration by Ft at the molecular level. For both L55P and F57S mutants, significant enhancement of cellular iron accumulation (Student's *t*-test, *P=*0.002, *n*=6 for L55P and *P*=0.003, *n*=6 for F57S) and molecular-level Ft iron loading (*P=*0.00003, *n*=6 for L55P and *P*=0.02, *n*=4 for F57S) were observed. Further gains could not readily be obtained by combining these mutations or by performing additional random mutagenesis on the isolated clones. The results nevertheless prove for the first time that intracellular Ft biomineralization processes can be engineered to produce substantial gains in iron accumulation by individual protein macromolecules.

The proteins were characterized to determine their stability and magnetic properties. Because melting of SPFt variants occurs only above boiling temperature, stability was examined by titration with the denaturant guanidinium hydrochloride (GdmHCl). Both wild-type SPFt and the three selected variants showed similar GdmHCl titration profiles in both native and iron-saturated states (Supplementary Fig. 3). This is consistent with the data of Fig. 2f, showing similar protein expression levels for each variant. Magnetization curves of the variants (0.1 mg ml^−1^ Fe) were determined by superconducting quantum interference device magnetometry at 5 K (Supplementary Fig. 4). All variants showed near-linear magnetization curves with minimal hysteresis and no evidence of saturation in the range from −10,000 to 10,000 Oe; this indicates that all the SPFt holomers are predominantly paramagnetic under the conditions assessed. A slight pinching in the −2,000 to 2,000 Oe range was observed for wild-type SPFt and mutant F57S, consistent with previously reported Ft magnetization curves^28^ and possibly indicative of ferrihydrite mineralization^29^. Approximate magnetic moments per mole of particles at 10,000 Oe were estimated in conjunction with the iron loading measurements from Fig. 2e, resulting in values of 90 (wild-type SPFt), 260 (L55P), 250 (F57S) and 160 (F123S) J/T.

### Mechanistic analysis of biomineralization by SPFt variants

**Figure 3:**
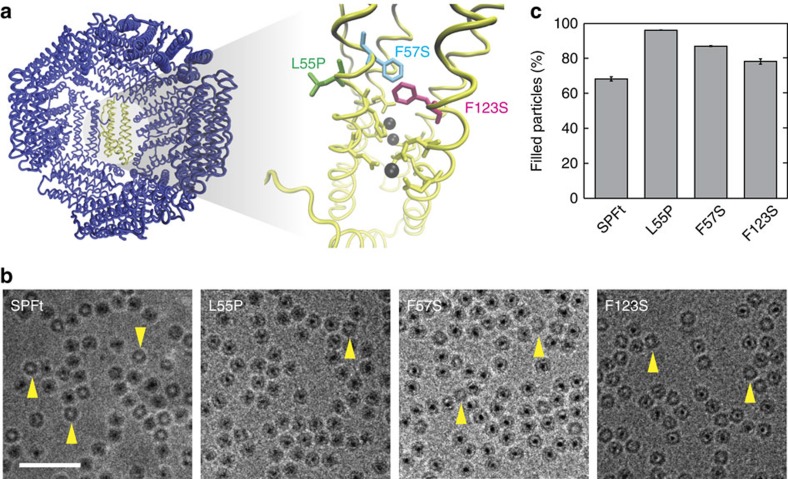
Structural analysis of SPFt variants. (**a**) X-ray crystal structure of PFt displaying the internal cavity of the protein in which one of the subunits is highlighted in yellow (left panel)^30^. Enlarged image of the highlighted subunit (right) shows the relative positions of sidechains mutated in the selected biomineralization mutants (L55P, F57S and F123S) with respect to the ferrooxidase residues (yellow) and the known iron binding sites (grey balls). (**b**) Cryo-EM images of purified SPFt, L55P, F57S, and F123S, showing formation of 12 nm spherically shaped nanoparticles in each case. SPFt samples also display differences in electron dense iron core formation, as indicated by the variable frequency of ‘empty' particles in the images (for example, yellow arrowheads). Scale bar, 50 nm. (**c**) The percentage of particles containing electron dense cores was computed by analysing 400 particles in cryo-EM images of each SPFt variant. All selected mutants displayed higher frequencies of core formation than the starting clone (*t*-test, *P*≤0.04), with L55P showing the greatest effect. Error bars denote s.e.m. of measurements from two independent samples.

In an attempt to understand the mechanism by which primary sequence mutations in SPFt lead to enhanced iron accumulation in the selected Ft holomers, we performed a series of characterization experiments. By inspecting the crystal structure of PFt^30^, we saw that all three mutant residues point towards the inside of the iron storage cavity and lie on the B and D helices close to a site thought to be involved in oxidation of Fe^2+^ ions that enter the PFt core (Fig. 3a). We speculated that the mutations might therefore affect either the enzymatic functionality of PFt or the structure of the iron oxide core itself. To test these ideas, we began by measuring the iron assimilation and release kinetics of the SPFt variants. No significant differences in iron oxidation (the initial step in core formation) or iron release rates were found (Supplementary Table 2).

To examine potential structural effects of the mutations, we characterized the purified protein nanoparticles by high-resolution cryo-electron microscopy (cryo-EM), a powerful technique that allows imaging of proteins in the near-native environment. Micrographs confirmed that SPFt and the variants all form 12 nm cage-like structures as expected (Fig. 3b). Image autocorrelation analysis indicated the presence of electron dense centres of 4–8 nm diameter for each variant (Supplementary Fig. 5). The L55P variant exhibited a marginally wider autocorrelation profile than other variants, possibly indicating a larger mean core size, while the F123S displayed the narrowest profile. There was more striking variation in the frequency of electron dense cores discernible among the four SPFt variants, however. Only 68.3±1.3% (mean±s.e.m.) of wild-type SPFt nanoparticles contained dark core structures, whereas 96.1±0.1%, 87.0±0.3% and 78.3±1.5% of the L55P, F57S and F123S mutants, respectively, appeared electron dense (Fig. 3c). Increased core formation in each mutant was significant with respect to SPFt (*t*-test; *P*=0.03 for L55P, *P=*0.04 for F57S, *P*=0.04 for F123S; *n*=2 samples with 400 particles per sample), suggesting that an increased ability of the mutant proteins to nucleate mineral core formation might largely account for their ability to accumulate a larger number of iron atoms per protein molecule. This explanation might also be compatible with the finding that the selected SPFt mutations could not be combined to further improve iron loading, given the possibility that enhanced mineral nucleation and growth at one site might not be compatible with nucleation directed at another site, and that competing nucleation and growth at multiple sites decreases homogeneity of mineral crystal formation^31^.

### Magnetic sorting and imaging using hypermagnetic SPFt

**Figure 4:**
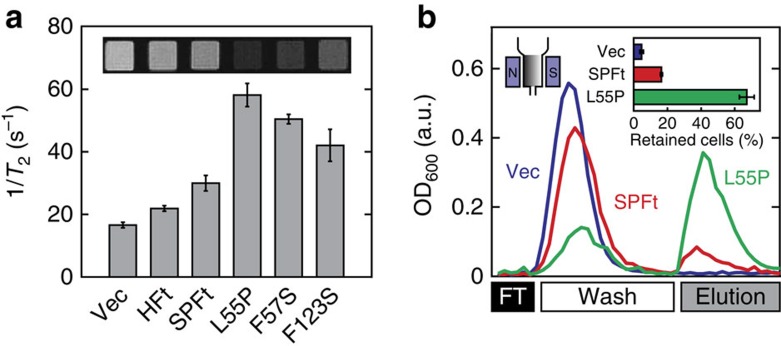
Engineered SPFt mutants are effective hypermagnetic probes in yeast. (**a**) Yeast cells transformed with empty vector (Vec), human heavy chain Ft (HFt), SPFt, L55P, F57S and F123S were pelleted and imaged in a 7 T MRI scanner. Relaxation rates (1/*T*_2_) were computed from the MRI signal amplitudes. Inset, corresponding *T*_2_-weighted spin echo MRI image of yeast cell pellets in microtiter wells (echo time=24 ms, repetition time=2,000 ms). (**b**) Isolation of yeast cells transformed with vector (blue), SPFt (red) and L55P (green) following application to a magnetic column. Cells were recovered during flow-through (FT), wash and elution phases of a magnetic cell separation protocol. Inset shows the percentage of cells retained until the elution phase, with L55P performing ∼4 times better than SPFt. Error bars denote s.e.m. of three independent measurements. a.u., arbitrary unit.

Our strategy for engineering hypermagnetic SPFt variants was predicated on the notion that iron sequestration by SPFt mutants would accompany enhanced magnetic properties. To demonstrate this, we explored the utility of hypermagnetic SPFt variants in imaging and high-gradient magnetic cell separation (HGMS) applications. For MRI experiments, the same yeast samples used for the iron assays in Fig. 2d were pelleted and imaged in a 7 T magnet using a spin-echo acquisition sequence. The transverse relaxation rate (1/*T*_2_) of cells transformed with the most iron-rich Ft mutant, L55P, was significantly higher than that of cells expressing wild-type SPFt (58.2±3.7 s^−1^ vs 30.0±2.5 s^−1^, *t*-test *P*=0.001, *n*=4) or human HFt (21.9±0.9 s^−1^, *P*=0.001, *n*=3), indicating that the hypermagnetic mutant L55P indeed shows higher sensitivity as an intracellularly expressed MRI contrast agent (Fig. 4a). The ability of SPFt L55P to enhance magnetic capture in HGMS was assessed by comparing the mutant protein to wild-type SPFt and Ft-free control cells. Yeast cells-expressing L55P were retained with four times greater efficacy than cells transformed with SPFt (Fig. 4b), demonstrating that the increased cellular magnetization due to expression of hypermagnetic mutant protein nanoparticles significantly improved the sensitivity of magnetic cell sorting process (*t*-test; *P*=0.007, *n*=3). HGMS and imaging results could not be explained by differences in the protein expression levels of wild-type SPFt versus the hypermagnetic mutant L55P; these variants expressed at similar levels of 0.45±0.03% and 0.53±0.07% of total soluble protein per cell, respectively. Moreover, the normalized transverse relaxation rates (1/*T*_2_ per mM Fe) of cells transformed with wild-type SPFt and the hypermagnetic mutants L55P, F57S and F123S were all very similar: 17.4±2.0, 21.5±1.9, 19.2±1.3 and 19.2±2.5 mM^−1^ s^−1^ for wild type, L55P, F57S and F123S, respectively. This indicates that the observed differences in magnetic behaviour are primarily due to the variation in the number of iron atoms accumulated in these cells, rather than to differences in per-iron relaxivity or magnetic moment.

**Figure 5:**
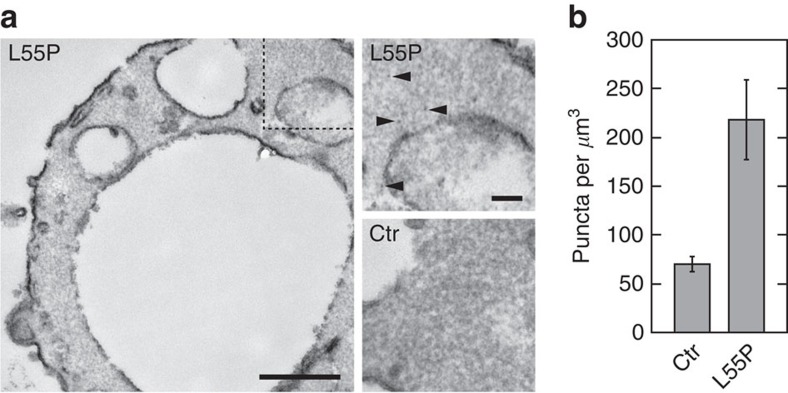
Detection of intracellular SPFt particles in ultrastructural cell images. (**a**) Representative TEM image of a yeast cell following transfection with SPFt L55P and growth in overnight in medium containing 1 mM ferric citrate before sample preparation (left, scale bar, 500 nm). A closeup of the region identified by the dashed box at left region shows electron dense puncta such as those indicated by arrowheads (top right, scale bar, 100 nm). Similar puncta are not apparent in a comparable region from control cells (bottom right). (**b**) Automated analysis of TEM images from SPFt-expressing (*n*=10) or control cells (*n*=4) enables quantification of puncta that correlate with coefficient ≥0.9 to a Gaussian spot with full width at half height of 7 nm, comparable to the expected SPFt mineral core size. The difference in the density of puncta in SPFt L55P-expressing versus control cells is significant with *t*-test *P*=0.05.

Because enhanced mineral accumulation and magnetism is explicitly associated with SPFt nanoparticles, as opposed to cellular mineral content more generically^9^,^10^,^32^, we hypothesized that the mutants identified here could provide means for engineering molecular-scale imaging markers and devices. At an ultrastructural level, SPFt mutants could for instance constitute effective genetically encoded labels for electron microscopic investigations of cells^18^. To address this possibility, we examined transmission electron microscopy (TEM) images of yeast transfected with SPFt L55P or with an empty control vector. Yeast expressing the SPFt variant showed distinct puncta of elevated electron density, each close in size to that expected for a Ft mineral core and visible on close examination of arbitrary cytosolic fields of view (Fig. 5a). Comparable images from control did not reveal similar puncta. As a quantitative indication of this difference, we used an automated template-matching procedure to identify approximately Gaussian electron-dense spots of 7 nm full width at half height in multiple TEM images of both SPFt-expressing and control yeast cells. Puncta that closely matched the template (correlation coefficient=0.9) were counted in cytosolic regions only (Fig. 5b). This analysis indicated a concentration of 220±40 puncta μm^−3^ from SPFt cell images (*n*=10) but only 70±8 puncta μm^−3^ from control images (*n*=4), a significant difference (*t*-test *P*=0.05) supporting the identification of these spots with SPFt nanoparticles. This suggests that SPFt variants could indeed function as TEM-detectable genetically encoded labels in engineered yeast and perhaps other cells.

### Construction of an intracellular sensor using SPFt L55P

**Figure 6:**
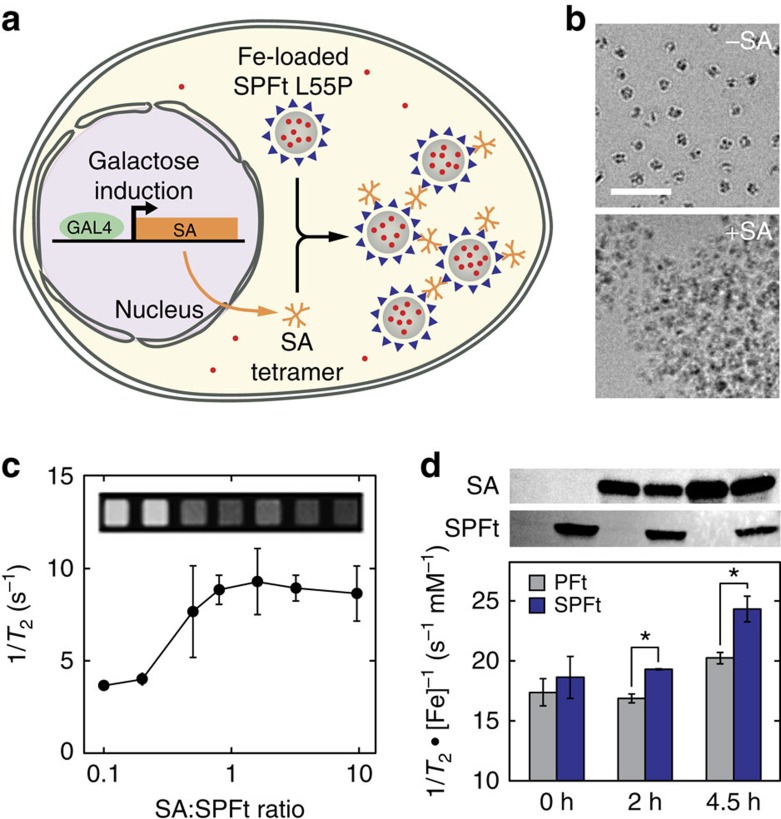
Construction of a SPFt-based intracellular magnetic biosensor. (**a**) Design of a genetically encoded magnetic biosensor for dynamic gene reporting using engineered SPFt. The sensor consists of two components: constitutively expressed SPFt L55P nanoparticles (grey) and streptavidin (SA) tetramers (orange) expressed from a galactose-inducible gene shown in the nucleus. When SA is upregulated, it crosslinks SPFt oligomers via their Strep-tag II moieties (blue), forming clusters that enhance *T*_2_ relaxation rates and provide a means for magnetic detection of galactose-induced SA expression. (**b**) Representative cryo-EM images showing aggregate formation by purified SPFt L55P in the presence (below) but not the absence (above) of SA. Scale bar, 50 nm. (**c**) SA-dependent changes in the relaxation rate (1/*T*_2_) displayed by solutions of 0.2 μM SPFt L55P holomers loaded with 520 μM Fe in the presence of increasing tetramer concentrations of an SA variant optimized for stability and Strep-tag II binding. Error bars show s.e.m. of three independent titrations, and the inset displays representative *T*_2_-weighted MRI images corresponding to conditions shown in the graph. (**d**) Magnetic detection of SA-mediated intracellular clustering of SPFt. SPFt L55P and galactose-inducible SA were coexpressed in yeast as schematized in **a**. After 16 h in galactose-free medium (10 mM Fe), cells were transferred to 2% raffinose media without iron for 2 h. Then galactose was added to the culture at a final concentration of 2% to induce SA expression and cells were harvested at 0, 2 or 4.5 h for analysis. *T*_2_ relaxation rates measured by MRI were normalized by cell pellet iron content and were compared with control experiments performed with PFt L55P (grey bars), which accumulates iron but is not crosslinked by SA. Normalized 1/*T*_2_ values at 2 h and 4.5 h were significantly higher in the SPFt-expressing cells (Student's *t*-test *P*=0.02–0.04, *n*=3), consistent with the mechanism in **a** and the results of **c**. Error bars indicate s.e.m. of three independent experiments and corresponding protein levels revealed by Western blotting against SA and the Strep-tag II moiety of SPFt are presented above.

In addition to potential utility for magnetic cell sorting, cellular MRI and electron microscopic investigations, the hypermagnetic SPFt variants are potentially useful building blocks for incorporation into magnetic molecular devices. As demonstration of this idea, we constructed a SPFt-based magnetic biosensor for galactose-induced gene expression in yeast (Fig. 6a). To design the biosensor, we made use of the dependence of magnetic relaxation properties on the aggregation state of iron-loaded Ft molecules^33^,^34^. Aggregation of purified SPFt L55P variant in buffer could be induced by mixing purified protein with streptavidin (SA) tetramers, which provide a multivalent partner for interactions with the Strep-tag II moiety on SPFt. Cluster formation could be observed at molecular scale by cryo-EM (Fig. 6b), and also resulted in visible precipitation and dynamic light scattering (DLS) changes (Supplementary Fig. 6). SA-mediated clustering could be promoted substantially by engineering SA for optimal folding and selective binding of Strep-tag II versus biotin. The SA/SPFt oligomer ratio required for half maximal clustering was 0.8 for optimized SA, but 60 for wild-type SA (Supplementary Fig. 7). In this respect, the optimized SA variant achieved maximal aggregation close to the conditions of 1:1 oligomer stoichiometry expected to optimize clustering under idealized conditions^35^. On the other hand, the large excess of wild-type SA required for SPFt clustering, which for the optimized SA would likely inhibit aggregation, probably reflects the inferior stability and SPFt-binding characteristics of the non-engineered SA. Titration of SPFt aggregation with optimized SA was measured by MRI, where a half maximal change in 1/*T*_2_ was observed at SA/SPFt ratio of 0.4 (Fig. 6c), consistent with the results from DLS.

Although Ft aggregation-based sensors have been demonstrated previously in cell-free solutions^33^,^34^, none has yet been reported to produce detectable responses on genetically directed intracellular expression. To demonstrate that this key step could be achieved using the magnetic molecular sensor formed by hypermagnetic SPFt and SA tetramers, a construct directing constitutive expression of the SPFt mutant was cotransformed with a galactose-inducible construct encoding optimized SA into yeast and grown in iron-supplemented medium overnight. Cells were transfered to galactose-containing medium to induce SA expression, followed with characterization by MRI, iron quantification and western blot analysis at time points 0, 2 and 4.5 h after the induction of SA overexpression (Fig. 6d). Relaxation rates were normalized by cellular iron concentration to factor out differences in iron accumulation over time, and results were compared with control experiments using PFt L55P, which lacks the ability to cluster with SA, in place of SPFt L55P. The SPFt variant displayed 7, 14 and 20% greater normalized 1/*T*_2_ than the PFt control at the 0, 2 and 4.5 h time points, respectively; differences at 2 and 4.5 h were significant with Student's *t*-test *P*=0.02 and 0.04, respectively. These results are consistent with the data showing increased relaxivity of SA-SPFt clusters versus unclustered SPFt in buffer. In contrast, no significant effects could be observed when SA expression was induced in the presence of SPFt K142R or PFt K142R mutants lacking iron-loading functionality (Supplementary Fig. 8), indicating that iron loading by the SPFt variants is required for relaxivity changes measured by MRI. These results together show that genetically encoded magnetic devices formed from SPFt mutants can be formed and applied inside cells. Such devices represent prototypes for a wide variety of noninvasive imaging sensors that could be expressed in cells, where they are likely to exhibit faster and more versatile responses than could be obtained by altering Ft expression itself^13^. Yeast bearing SPFt-based magnetic devices might also be applicable as whole-cell-based sensors in opaque media or living organisms.

## Discussion

In this report, we have shown that a high-throughput protein selection strategy can be applied to enhance intracellular molecular-level biomineralization within Ft variants, resulting in proteins with the ability to induce magnetic phenotypes, influence imaging signals at multiple scales and serve as building blocks for intracellular magnetic devices. Mechanistic analysis of the SPFt mutants identified here indicated that single amino acid substitutions significantly enhanced the uniformity of mineral formation within SPFt expressed in yeast. This result could not have been predicted from the PFt structure alone, validating the random library construction approach we took, and also shows that screening for iron sequestration phenotypes can complement traditional site-directed mutagenesis studies^36^,^37^,^38^ to expand knowledge about the mechanisms of iron mineralization by Ft.

The specific mutagenesis and screening approach taken here is one of a universe of approaches that could have been taken to obtain desirable Ft biomineralization mutants. Selecting for iron accumulation as opposed to magnetic properties biased the screen towards variants with increased mineral core formation (Fig. 3 and Supplementary Fig. 5), potentially enhancing the performance of selected variants as TEM labels (Fig. 5). For magnetic applications such as imaging and magnetic sorting (Fig. 4), as well as magnetic biosensing (Fig. 6), screening directly for magnetic properties might have been more direct, but magnetic assays tend to be both less sensitive and cruder. For instance, our own initial experience with magnetic column-based selection approaches (*cf.* Fig. 4b) revealed several sources of artifacts, ranging from nonspecific column adhesion to cell clumping, which would compromise the efficacy of a screen. Although further improvement in magnetic screening techniques is certainly possible in the future, indirect screening using optical approaches proved useful here, as in our earlier work with magnetically active MRI sensors^39^. With either type of approach, careful molecular analysis of selected clones must be performed to avoid mutations unlinked to the desired phenotype (such as changes in clonal growth rate), but nevertheless artificially favored by the screening technique.

Although all of our experiments were performed in yeast, the protein engineering principles applied here and potentially the specific clones identified could be applied for biotechnological applications in other eukaryotic or prokaryotic cellular environments. Further optimization could be necessary to enhance Ft variant expression in the desired host, or to adapt the magnetic proteins to iron homoeostasis and protein folding conditions in other systems. In any context, manipulating mineral nucleation could prove to be a general and versatile route for tuning intracellular biomineralization, particularly if unnatural mineral species are desired^40^. Protein engineering approaches like those introduced here could also be used to engineer additional metalloproteins, and could further alter other parameters of genetically expressed magnetic biomaterials and biosensors.

## Methods

### Yeast strain and handling methods

We used the haploid yeast (*S. cerevisiae*) strain BY4742/*FTR1–GFP* (MAT α *FTR1–GFP::HISMX* his3Δ1 leu2Δ0 lys2Δ0 ura3Δ0)^25^ (gift from Dr Christopher Burd) as a host for expression of all Ft variants. We grew yeast cells in a dropout medium without histidine (SD-HIS) made with a dry culture medium (Teknova, Hollister, CA) or in a YPAD medium: 10 g l^−1^ yeast extract (BD Biosciences, San Jose, CA), 20 g l^−1^ of Bacto Peptone (BD Biosciences), 20 mg l^−1^ of adenine hemisulfate and 20 g l^−1^ glucose. We transformed yeast cells with expression plasmids using the Frozen-EZ Yeast Transformation II kit (Zymo Research, Irvine, CA).

### Construction of Strep-tag II/ferritin fusion proteins

We used *Escherichia coli* NEB10β cells (New England Biolabs, Ipswich, MA) for plasmid construction. To create an expression plasmid with a dominant selectable marker, we used the PCR to amplify a geneticin resistant cassette, KanMX4 from a plasmid pFA6-kanMX4^41^ kindly provided by Dr Peter Philippsen. We subcloned the PCR product containing KanMX4 fragment into the pHVX2 yeast expression plasmid generously supplied by Dr Hennie Van Vuuren^42^. We then made a point deletion to destroy a superfluous *Eco*RI site by the QuikChange Lightning Kit (Agilent Technologies, Santa Clara, CA) to yield the host plasmid, pHVX2G, used for subsequent expression of Ft constructs in our experiments. We amplified ferritin gene of PFt from the genomic DNA of the bacteria (ATCC, Manassas, VA). A Strep-tag II sequence (WSHPQFEK), spacer (GTSS) and restriction sites were genetically fused at the 5′-end of the PFt gene and the PCR product was subcloned into pHVX2G to yield plasmid pHVX2G-SPFt (Supplementary Table 3).

### SPFt expression and affinity purification

For expression of SPFt, we inoculated yeast cells with expression plasmids in 1 ml of YPAD media with 200 μg ml^−1^ Geneticin and incubated overnight at 30 °C. We then diluted the cultures into fresh media at OD_600_∼0.04 and incubated them for 16 h at 30 °C before harvesting. We washed the freshly harvested yeast with 30 ml of PBS+10 mM EDTA twice and finally resuspended in PBS. We lysed yeast cell pellet with Y-PER Plus (Thermo Scientific, Waltham, MA), benzonase nuclease (EMD Millipore, Billerica, MA) and protease inhibitors according to the manufacturer's instructions. We then centrifuged the lysate at 3,000 *g* for 20 min at 4 °C. SPFt protein was purified by applying the cleared lysate into the Strep-Tactin sepharose column (IBA, Goettingen, Germany) according to the manufacturer's instructions, except EDTA that was omitted from the wash and the elution buffers. We buffer exchanged and concentrated the purified protein into the wash buffer using a spin filter with 100 kDa cutoff membrane (EMD Millipore). We measured the protein concentrations by the Pierce 660 nm Protein Assay (Thermo Scientific), with bovine serum albumin (BSA) as a standard.

### TEM of purified SPFt

For conventional TEM, we applied 1–3 μl of 0.05 mg ml^−1^ SPFt sample onto a carbon/copper-coated grid (Electron Microscopy Sciences, Hatfield, PA), removed the excess solution with a filter paper and let it dry for 30 s. We then applied 15 μl of 1% phosphotungstic acid (pH 7.0) over the sample for about 10 s and removed the excess stain with a filter paper. The grid was dried at room temperature for at least 1 h before imaging with a JEOL 2010 HRTEM instrument (JEOL, Tokyo, Japan).

For cryo-EM, we applied 5 μl of the protein and buffer solution on a lacey copper grid coated with a continuous carbon film and removed excess sample without damaging the carbon layer using a Gatan Cryo Plunge III (Gatan, Pleasanton, PA). We mounted the grid on a Gatan 626 cryo-holder equipped in the TEM column and kept it under liquid nitrogen throughout the transfer into the microscope and the subsequent imaging session. We imaged the SPFt samples on a JEOL 2100 FEG microscope (JEOL) using a minimum-dose method that was essential to avoid sample damage under the electron beam. We imaged at 200 kV with a magnification setting of 60,000 × for assessing particle size and distribution and recorded the images on a Gatan 2k × 2k UltraScan CCD camera (Gatan, Pleasanton, PA).

To calculate the percentage of filled cores, we counted 400 particles per sample and divided the number of filled particles by 400. For each SPFt variant, we obtained cryo-EM images of the protein samples from two different batches to calculate mean, s.e.m., and statistical parameters. To estimate core sizes, autocorrelation functions were computed from the same TEM images (three per variant) in Matlab, generating autocorrelation plots and radial profiles presented in Supplementary Fig. 5.

### Library construction

We carried out library construction using an error-prone PCR approach^43^. The entire SPFt gene except for the Strep-tag II sequence was subjected to mutagenesis over 30 error-prone amplification cycles, which yielded on average one amino acid mutation per SPFt gene. The linearized vector was prepared by digesting pHVX2G with *ApaI* and *XhoI* followed by gel purification. We transformed yeast with the SPFt library according to the method developed by Benatuil *et al*. with a few modifications. We mixed 1.5 μg of digested plasmid and 0.5 μg of error-prone PCR product with 100 μl of electrocompetent cells (∼1.6 × 10^9^ cells per ml) in a disposable electroporation cuvette with 0.2 cm gap (Bio-Rad, Hercules, CA) on ice for 5 min. We electroporated the cells at 3 kV using MicroPulser electroporator (Bio-Rad), resulting in time constants ranging from 4.8 to 5.3 ms. After electroporation, we immediately transferred the cells to 1:1 mix of 1 M sorbitol:YPAD medium and incubated in 30 °C for 3 h. We then harvested cells by centrifugation and resuspended in SD-HIS with 200 μg ml^−1^ of Geneticin and incubated for 2 days before freezing them for long-term storage at −80 °C. Typical transformation efficiency was 0.5–1.0 × 10^7^ transformants per μg of plasmid DNA. The library diversity was tested by sequencing randomly picked 24 colonies.

### Measurements of iron content in cells and purified protein

We used a colorimetric assay based on the protocol of Tamarit *et al*.^44^ to quantify the iron content of yeast cells and the purified protein. This method relies on the Fe^2+^-dependent optical absorbance of bathophenanthrolinedisulfonic acid (BPS) at 535 nm at pH 5.4. As standards, we dissolved known amounts of ferrous ammonium sulfate in 3% nitric acid.

For measuring the iron content of yeast cells, we digested 4.2 × 10^8^ cells by boiling in 200 μl of 3% nitric acid for 2 h, and centrifuged at 10,000 *g* for 5 min. To measure the concentration of iron in SPFt, a 1:1 ratio of purified protein and 3% nitric acid solution were mixed and boiled for 15 min followed by centrifugation at 10,000 *g* for 5 min. In both cases, the iron quantification assay was applied to the supernatant of the resulting samples. Iron loading stoichiometries of the protein samples were computed by dividing the iron concentrations by the protein concentrations, as measured by the 660 nm Protein Assay (Thermo Scientific).

### High-throughput screening

We inoculated 1 × 10^8^ cells in a 20 ml SD-HIS medium containing 200 μg ml^−1^ of Geneticin at 30 °C overnight (about 16–20 h). We harvested the cells in a culture tube and resuspended in a sterile PBS such that the cell density was about 5 × 10^7^ cells per ml. We filtered the cells with a sterile membrane with 40 μm pores immediately before sorting. Similarly, we prepared negative control samples using the BY4742 background strain without the FTR1–GFP reporter. We set up a flow cytometry protocol using the control yeast samples. First, the yeast population was gated with forward and side scattering channels to remove debris and aggregated cells. We then collected cells displaying green fluorescence in the top ∼5%, indicating high FTR1-GFP expression. We propagated these cells overnight in 4 ml of SD-HIS medium supplemented with 200 μg ml^−1^ of Geneticin.

### Measurement of iron oxidation and release kinetics

We monitored the kinetics of iron oxidation by SPFt variants by an optical assay^45^. We prepared SPFt samples with 100 Fe/24-mer in 100 mM MOPS, pH 7.0. We added ferrous ammonium sulfate solution (1 mM), made in degassed distilled water to the protein solution (final concentration of 0.1 μM) at a 500-fold molar excess of iron(II). Following a mixing dead time (∼5 s), we recorded the optical absorbance of the mixture at 315 nm every 2 s for 5 min. We used a disposable cuvette with a 1 cm path length and recorded the spectra with SpectraMax M2 Microplate reader (Molecular Devices, Sunnyvale, CA). We calculated the specific activity, defined as the micromoles of iron(III) formed per minute per milligram of 24-mer SPFt by dividing the change in absorbance of the reaction mixture over the first 30 s by the extinction coefficients of SPFt variants and the amount of protein in the reaction. Extinction coefficients for wild type SPFt, L55P, F57S and F123S were 2.6±0.1, 2.6±0.1, 2.7±0.1 and 2.8±0.1 mM cm^−1^, respectively.

We measured the kinetics of iron release from preloaded SPFt variants by monitoring time dependent formation of the BPS complex with Fe^2+^ released from iron-loaded Ft variants. We used purified SPFt samples that were loaded aerobically with 1,000 Fe atoms per molecule. These samples were diluted to a final concentration of 0.1 μM SPFt oligomers in an iron mobilization assay buffer that included MOPS (0.1 M, pH 7.0), sodium acetate (20 mM) and BPS (1 mM). We measured the absorbance values at 535 nm every 30 s for 3 h using SpectraMax M2 Microplate reader. We took the first 3.5 min of the data and computed the initial rate of iron release using the standard curve constructed using freshly made ferrous ammonium sulfate solutions.

### Measurement of magnetization curves

100 μl of each SPFt variant dispersed in Tris buffer (0.1 mg ml^−1^ Fe) was sealed in a propylene straw using a hot press. Zero field cooled curves at 5 K were measured using a superconducting quantum interference device (MPMS/XL, Quantum Design, San Diego, CA). Diamagnetic background signal was subtracted by measuring a buffer only sample sealed in the same manner.

### Measurement of denaturation profiles

SYPRO dye (Life Technologies) was diluted 25-fold from the manufacturer's 5,000 × stock into 0.1 M Tris, 0.15 M NaCl, pH 8.5. 1.5 μl of each SPFt variant (0.39 mg ml^−1^) was mixed with 1.5 μl SYPRO solution and 27 μl GdmHCl to a final concentration of 0–8 M GdmHCl. After 10 min incubation at room temperature, fluorescence intensity was measured using a plate reader with excitation at 567 nm and emission at 580 nm.

### Yeast cell pellet MRI

We prepared the yeast samples as described in SPFt expression and purification section. After we washed the cells twice with PBS supplemented with 10 mM EDTA, the supernatant was decanted and 100 μl of the cell suspension was loaded into the wells of a microtiter plate. Unused wells were filled with PBS. We centrifuged the plate at 1,500 *g* for 3 min and placed it in a 12 cm outer diameter birdcage transceiver for imaging in a 20-cm bore Bruker 7 T Avance III MRI scanner. We imaged a 2 mm slice through the cell pellet samples with the field of view of 5 × 5 cm and the data matrices were 256 × 256 points. We used *T*_*2*_-weighted spin echo pulse sequence with multiecho acquisition; repetition time was 2 s, and echo time ranged from 5 to 150 ms in 5 ms intervals. We used custom routines written in Matlab (Mathworks, Natick, MA) to reconstruct the images and computed relaxation time constants by fitting image intensity data to exponential decay curves.

### Magnetic cell sorting

High gradient magnetic separations of yeast cells were performed using magnetic columns (Miltenyi Biotec, Bergisch Gladbach, Germany) inserted into a Frantz Canister Separator, Model L-1CN (S. G. Frantz Company, Inc., Tullytown, PA). Briefly, we suspended yeast cells at the density of 2 × 10^8^ cells per ml in a sorting buffer consisting of PBS supplemented with 2 mM EDTA and 0.5% BSA. After equilibrating the column with the sorting buffer, we applied the yeast cells on the column in the presence of an externally applied magnetic field of 0.6 T followed by a wash with the sorting buffer. We then switched off the magnetic field and eluted the cells from the column with the sorting buffer. We collected the flow through, the wash and the elution fractions from the column into a 96-well microtiter plate. We carried out optical density measurements at 600 nm to estimate the cell densities of each fraction and computed the percentages of cells retained on the columns.

### Electron microscopy analysis of SPFt particles in cells

For electron microscopy, yeast cells were grown in YPD medium supplemented with 1 mM ferric citrate overnight. Cells were then harvested, washed in PBS and spheroplasted before transferred to a fix buffer (3% glutaraldehyde, 0.1 sodium cacodylate, 5 mM CaCl_2_, 5 mM MgCl_2_, 2.5% sucrose). Cells were embedded in 2% ultralow melting temperature agarose and cut into small pieces. Sample blocks were post-fixed in 1% osmium/0.1% potassium ferrocyanide in 0.1 M cacodylate and 5 mM CaCl_2_ for 30 min at room temperature. Sample blocks were washed thoroughly and transferred to 1% thiocabohydrazide at room temperature for 5 min followed by another wash. The sample blocks were transferred to 1% osminum/1% potassium ferrocynanide in cacodylate buffer for 5 min at room temperature followed by another wash. The sample blocks were then dehydrated in increasingly concentrated ethanol solutions and embedded in Spurr resin. Blocks were sectioned on a Leica Ultracut UCT (Leica Microsystems Inc., Buffalo Grove, IL), stained with 2% uranyl acetate and imaged using FEI Tecnai Spirit transmission electron microscope at 80 kV (FEI, Hillsboro, OR).

To quantify putative Ft particles, images were first manually segmented to define cytosolic compartments, in particular by excluding membrane, extracellular space and vacuoles. A template for matching to the images was defined by specifying a dark Gaussian spot of 7 nm full width at half height on a white background of 20 × 20 nm. This template was then compared with the images using Fourier-based correlation, to identify image locations that displayed correlation coefficients of 0.9 or greater when matched to the template. Groups of one or more contiguous pixels were counted as a single particle. A total of 10 images of SPFt L55P-transfected cells and 4 images of control cells were analysed in this way and results were scaled to denote the concentration of qualifying puncta per cubic micron. This analysis was performed using custom code implemented in Matlab.

### Construction of T7-tagged mutant streptavidin plasmids

We used the PCR with High-Fidelity Phusion master mix (New England Biolabs) to construct the gene of an SA variant optimized for efficient folding and selective binding of Strep-tag II (Supplementary Table 4). The optimized SA mutant contains N-terminal T7 tag and four mutations (E44V, S45T, V47R and W120A). We used a plasmid, pSA1 T7SA W120A (a gift from Dr Blake Peterson)^46^ as a template for an inverse PCR to introduce the following three mutations (E44V, S45T and V47R) to obtain a new plasmid, pSA1 STm. We amplified the gene encoding mutant SA from pSA1 STm and subcloned it into NdeI/EcoRI sites of an *E. coli* expression plasmid, pT7-7 (a gift from Dr Nicholas Reiter), resulting in the pT7-7 STm vector. A yeast expression plasmid encoding the new mutant SA was constructed with Zeocin as a selection marker, suitable for coexpression with SPFt in yeast. We amplified the 1.2 kb fragment containing the Zeocin resistance cassette from pPICZA (Life Technologies, Carlsbad, CA), digested with *Bst*BI and *Aat*II, and cloned into pSA1 T7SA to replace TRP1 marker, thereby producing the pSAZ T7SA plasmid. The gene encoding optimized SA was amplified from pSA1 STm, digested with *Nhe*I and *Xho*I and cloned into pSAZ T7SA to replace T7SA with the mutant *SA* gene and yield the pSAZSTm plasmid.

### Bacterial expression and purification of SA variants

To express SA variants, we transformed *E. coli* with the plasmid, pT7-7 STm and grown in M9 minimum medium supplemented with 100 μg ml^−1^ ampicillin at 37 °C. Once the culture reached OD_600_∼0.8, we induced the recombinant protein expression with 0.4 mM isopropyl β-D-1-thiogalactopyranoside (IPTG) for 4 h at 30 °C. We harvested and lysed cells with BugBuster reagent (EMD Millipore) supplemented with protease inhibitor cocktail III (EMD Millipore) and Lysonase Bioprocessing Reagent (EMD Millipore) for 30 min at room temperature. Insoluble fractions were removed by centrifugation at 10,000 *g* for 40 min. The soluble fraction of lysate was used for the affinity purification of optimized SA using T7-Tag Affinity Purification Kit (EMD Millipore) according to the manufacturer's instructions. We then buffer exchanged the purified protein and concentrated into the assay buffer. Wild-type SA was purchased from Sigma-Aldrich (St Louis, MO). Protein concentrations were determined using the 660 nm Protein Assay (Thermo Scientific) with BSA as a standard.

### DLS measurements

We performed DLS measurements on a DynaPro DLS system (Wyatt Technology, Santa Barbara, CA), at 30 °C with averaging over 72 acquisitions each and a 2 s integration time. The laser power was set to 25%. We mixed 16 μl of 0.2 μM 24-mer SPFt sample with various concentrations of SA tetramers, briefly vortexed, and incubated for 5 min before making the DLS measurements in triplicates.

### Coexpression of SPFt variants and SA in yeast

To test the SPFt-based biosensing system in cells, we transformed yeast with two expression plasmids, pHVX2G-SPFt-L55P and pSAZSTm encoding SPFt L55P or optimized mutant SA, respectively. Control experiments were performed using plasmids encoding PFt L55P, SPFt E94G/K142R, or PFt E94G/K142R in place of SPFt L55P. We first incubated the yeast cells in a rich medium with 2% glucose and 10 mM ferric citrate overnight to allow SPFt or PFt expression and iron loading. We then transferred the yeast cells into 2% raffinose plus 0.1% glucose medium and incubated for 2 h. We then induced expression of SA by adding galactose at a final concentration of 2% and harvested cells at 0, 2 and 4.5 h time points to measure expression levels and make MRI and iron content measurements from cell pellets.

### Western blot analysis of yeast cell pellets

For western blotting experiments, the whole cell lysate samples were prepared from yeast cells freshly harvested after overnight incubation according to the method developed by von der Haar with a few modifications^47^. Equivalent numbers of yeast cells (2.1 × 10^8^) were resuspended in 100 μl of the lysis buffer and boiled for 10 min. The cell suspensions were neutralized by the addition of 2.5 μl of 4 M acetic acid, vortexed for a minute, and boiled for another 10 min. We then added 25 μl of the loading buffer to the samples and centrifuged them at 10,000 *g* for 5 min before loading onto a 12% Mini-Protein TGX Precast gel (Bio-Rad). We ran the protein gels at 160 V for 30 min and transferred the separated proteins onto PVDF membranes (Bio-Rad) at 100 V for 40 min at 4 °C. The membranes were blocked with 5% fat-free milk in Tris-buffered saline (AMRESCO, Solon, OH) containing 0.1% Tween 20 (TBST) for 30 min at 4 °C. For visualization of SPFt variants, we washed the membranes once with TBST for 5 min and incubate them with Strep-Tactin-horseradish peroxidase conjugate (IBA, Goettingen, Germany) at 1:4,000 dilution in TBST for 1 h at room temperature. For imaging expression of SA, we used an anti-streptavidin antibody conjugated to horseradish peroxidase (Abcam, Cambridge, UK) at 1:10,000 dilution in TBST. After washing the membranes three times with TBST, we visualized the SPFt and SA bands with a chromogenic substrate Opti 4CN (Bio-Rad) according to the manufacturer's instructions. Images of the membranes were taken and processed with ImageJ software for quantitative analysis.

## Additional information

**How to cite this article:** Matsumoto, Y. *et al*. Engineering intracellular biomineralization and biosensing by a magnetic protein. *Nat. Commun.* 6:8721 doi: 10.1038/ncomms9721 (2015).

## Supplementary Material

Supplementary Information Supplementary Figures 1-8 and Supplementary Tables 1-4
