# Supplementary material for: Engineering intracellular biomineralization and biosensing by a magnetic protein
Source: Nat Commun. 2015 Nov 2;6:8721. doi: 10.1038/ncomms9721 (PMC4667635; doi:10.1038/ncomms9721)
Supplement: Supplementary Information — Supplementary Figures 1-8 and Supplementary Tables 1-4 [file ncomms9721-s1.pdf]

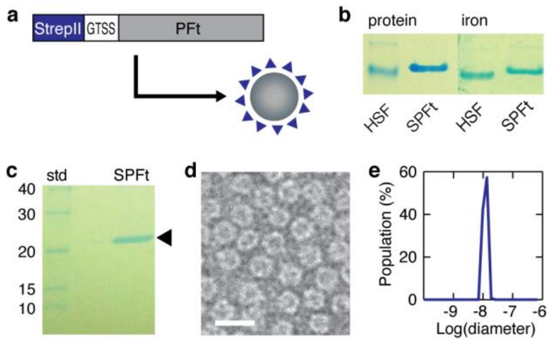

Supplementary Figure 1. Design and characterization of affinity-tagged PFt

a, Schematic of the DNA construct leading to self-assembled SPFt. Each polypeptide chain contains an N-terminal Strep-tag II (blue), a GTSS linker (white), and the Ft gene from *Pyrococcus furiosus* (gray); these proteins form homooligomers of 24 subunits each. **b**, Native gel analysis of horse spleen ferritin (HSF), containing approximately 3,000 Fe atoms per 24-mer, and purified wild type SPFt, aerobically loaded with 1,500 Fe/24-mer, together stained with Coomassie blue (left) and Prussian blue (right). The Prussian blue stain indicates iron content semiquantitatively, and shows that SPFt is capable of loading iron *in vitro*. **c**, Coomassie-stained sodium dodecylsulfate polyacrylamide gel showing that affinity purification yields highly pure SPFt, with a single band near the expected molecular weight of 22 kD indicated by a black arrowhead. Molecular weight standards (std) are shown at left. **d**, Transmission electron micrograph of SPFt with negative staining showing iron mineral cores surrounded by protein shells of about 12 nm in diameter, consistent with the crystal structure of this PFt. Scale bar = 20 nm. **e**, Dynamic light scattering size histogram of SPFt nanoparticles, showing an average hydrodynamic diameter of about 12 nm.

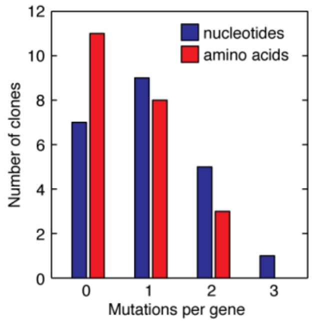

Supplementary Figure 2. Mutation rates in the initial SPFt library

Distribution of the number of nucleotide (blue) and amino acid (red) mutations per gene in the SPFt library used as the starting point for screening. The average number of DNA-level mutations is 1.0 and the average number of coding changes is 0.6.

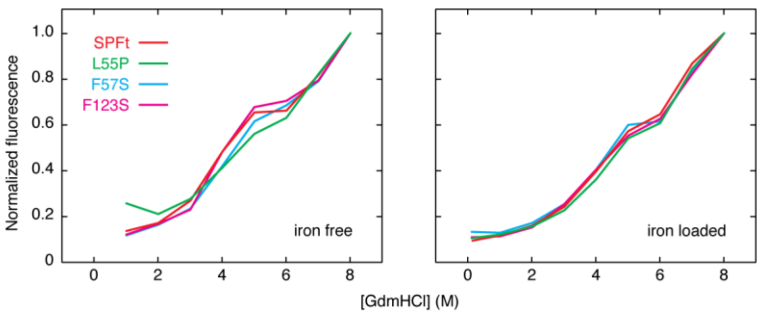

Supplementary Figure 3. Stability of wild-type SPFt and selected mutants

Stability of wild-type SPFt and selected mutants was analyzed by examining fluorescence changes in the presence of the dye SYPRO Orange upon titration with the denaturant guanidinium hydrochloride (GdmHCl) over a range from 0 to 8 M. Data were obtained from SPFt variants either without (left) or with (right) pre-treatment to saturate iron content; in each case 10 min. incubation at the stated GdmHCl concentration was performed prior to measurement. Denaturation of protein at increasing GdmHCl concentrations results in greater dye binding and increasing fluorescence. All variants under both conditions show evidence of a bimodal transition with an intermediate asymptote or inflection point between 5-6 M GdmHCl. Initial denaturation transitions occur with midpoints of ~4 M GdmHCl, and indicate similar stability for all variants. For comparison, horse spleen Ft denatures with a midpoint below 3 M GdmHCl (data not shown).

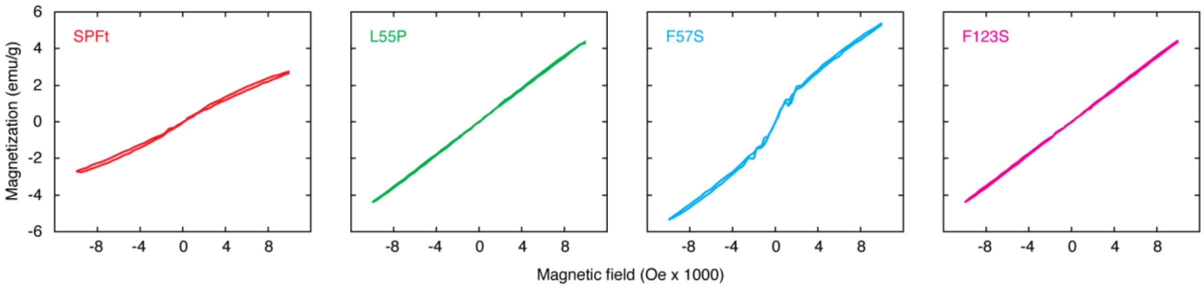

Supplementary Figure 4. Magnetization of wild-type SPFt and selected variants

Magnetization of SPFt samples was measured as a function of magnetic field using a superconducting quantum interference magnetometer operated at 5 K over a range from -10,000 to 10,000 Oe. Data were obtained for wild-type SPFt and mutants L55P, F57P, and F123S. Magnetization curves are similar and reveal paramagnetic behavior with little evidence of saturation or hysteresis at the fields examined. Data from wild-type SPFt and F57 show a slight pinching in the range from -2,000 to 2,000 Oe, similar to previously reported Ft magnetization curves.

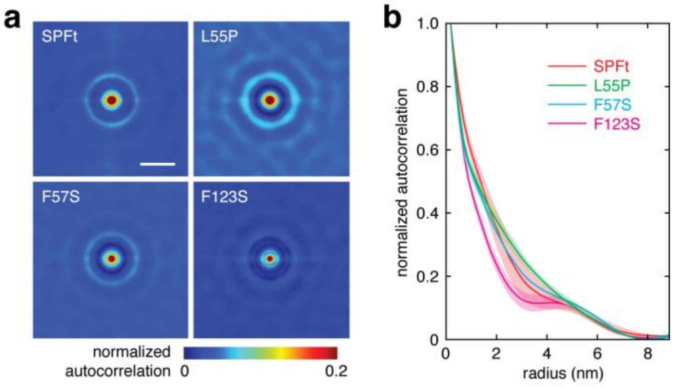

Supplementary Figure 5. Core size distribution in SPFt electron microscopy images

a, Core size distributions were estimated by computing intensity autocorrelation functions from cryo-EM images as in Figure 3c. Scale bar = 10 nm. **b,** Graphs of the intensity autocorrelation as a function of radius for wild-type SPFt and three selected mutants show slight differences in width. The widest autocorrelation function is exhibited by the L55P mutant, possibly indicated a larger mean core diameter. The narrowest autocorrelation function is exhibited by the F123S mutant. Error margins (shading) indicate standard error over autocorrelation functions computed independently from three cryo-EM images per SPFt variant.

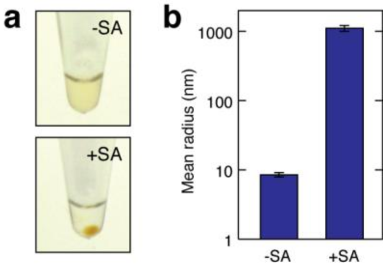

Supplementary Figure 6. SA-induced aggregation of SPFt L55P

a, Samples containing 0.2 µM of affinity purified 24-mer SPFt L55P were centrifuged for 10 s at 14,000 *g* within seconds following addition of 20 µM tetrameric SA or vehicle, resulting in pellet formation in the presence (bottom) but not the absence (top) of SA. **b,** Cluster formation by SPFt and SA was visible by dynamic light scattering. The mean radius of particles in 0.2 µM SPFt L55P solution increased by a factor of ~100 in the presence of 20 µM SA with respect to the –SA control. Error bars denote s.e.m. of three measurements.

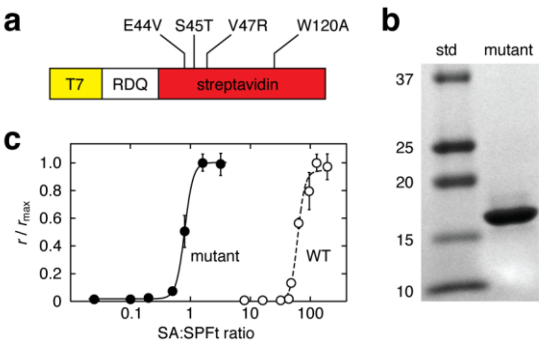

Supplementary Figure 7. SA optimized for biosensing with SPFt

a, Schematic of the DNA construct of the SA variant optimized for intracellular crosslinking of SPFt oligomers in yeast. The construct includes an N-terminal T7 tag fused to SA via a three amino acid RDQ linker. Mutations in the SA coding region reduce biotin binding and improve specificity for Strap-tag II (Voss, 1997). **b,** A Coomassie blue-stained sodium dodecylsulfate polyacrylamide gel indicating purity and expected size of optimized SA obtained by bacterial expression (right lane), compared with standard markers (left lane). **c,** Mean particle size as measured by dynamic light scattering in solutions containing 0.2 μM purified SPFt L55P holomers as a function of the stoichiometric ratio of tetrameric wild-type (WT) or optimized mutant SA to SPFt 24-mers. Curves indicate best fits to a four-parameter logistic function. Error bars indicate s.e.m. of three measurements.

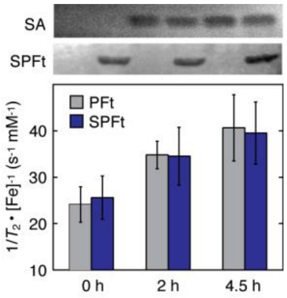

Supplementary Figure 8. Biosensing is unobserved with iron-deficient SPFt analogs

Control experiments conducted with a nonmagnetic mutant version of SPFt containing E94G and K142R substitutions show that iron loading is required for biosensing in the SPFt/SA system of Fig. 5. Mutant SPFt nanoparticles were constitutively expressed in yeast while SA expression was induced by galactose; protein levels at 0, 2, and 4.5 hours post-induction are indicated in Western blot images in the top panel. There were no significant differences at any time point in normalized relaxation rates ($1/T_2 \bullet [Fe]^{-1}$) displayed by SPFt E94G/K142R-expressing cells (blue bars) vs. cells expressing PFt E94G/K142 (gray bars), which lacks both iron loading and SA binding functionalities. Error bars indicate s.e.m. of three independent experiments.

Supplementary Table 1. Distribution of mutations in selected SPFt clones*

	Identified mutations																				
clone	M1N	S3G	M6T	N11D	Y24C	E49G	L55P	F57S	Y58C	I61T	E63G	R67G	I97V	K99E	F123S	E129G	E130K	S133R	K135R	K139R	A162V
1						X						X									
2															X						
3																	X				
4							X														
5							X														
6							X			X											
7								X													
8						X															
9								X													
10							X			X											
11						X			X												
12				X											X						
13																X					
14														X	X				X	X	
15							X			X											
16							X			X											
17																					
18		X											X					X			
19			X		X																
20						X															
21						X															X
22							X			X											
23							X			X											
24								X			X										
25					X																
26					X																
27							X			X											
28							X				X										
29								X													
30	X																				
31				X											X						

* Crosses denote mutations discovered by sequencing SPFt in each of 31 clones selected following four rounds of cell sorting. L55P, F57, and F123S (magenta) were discovered with frequencies of 10, 4, and 4, respectively. Y24C and E49C (cyan) were also found in multiple clones, but did not display greater iron loading than WT SPFt. Mutation I61T (green) was identified in multiple clones, but only in combination with L55P. This mutation did not appear to enhance iron loading compared with L55P, but it decreased expression and was therefore omitted from further experiments.

Supplementary Table 2. Kinetics of iron oxidation and release by SPFt variants*

Ferritin variant	Iron oxidation specific activity (U/mg)	Iron release initial rate (μM/min.)
SPFt	0.22 ± 0.03	0.44 ± 0.04
L55P	0.26 ± 0.01	0.39 ± 0.00
F57S	0.31 ± 0.01	0.54 ± 0.02
F123S	0.28 ± 0.03	0.43 ± 0.01

*Reported values reflect mean and standard error of three independent measurements.

Supplementary Table 3. DNA sequence of plasmid pHVX2G-SPFt

GCGCCCAATACGCAAACCGCCTCTCCCCGCGCGTTGGCCGATTCATTAATGCAGCTGGCACGACAG
GTTTCCCGACTGGAAAGCGGGCAGTGAGCGCAACGCAATTAATGTGAGTTAGCTCACTCATTAGGCA
CCCCAGGCTTTACACTTTATGCTTCCGGCTCGTATGTTGTGTGGAATTGTGAGCGGATAACAATTTCA
CACAGGAAACAGCTATGACCATGATTACGCCAAGCTTTCTAACTGATCTATCCAAAACTGAAAATTAC
ATTCTTGATTAGGTTTATCACAGGCAAATGTAATTTGTGGTATTTTGCCGTTCAAAATCTGTAGAATTT
TCTCATTGGTCACATTACAACCTGAAAATACTTTATCTACAATCATACCATTCTTATAACATGTCCCCTT
AATACTAGGATCAGGCATGAACGCATCACAGACAAAATCTTCTTGACAAACGTCACAATTGATCCCTC
CCCATCCGTTATCACAATGACAGGTGTCATTTTGTGCTCTTATGGGACGATCCTTATTACCGCTTTCA
TCCGGTGATAGACCGCCACAGAGGGGCAGAGAGCAATCATCACCTGCAAACCCTTCTATACACTCA
CATCTACCAGTGTACGAATTGCATTCAGAAAACTGTTTGCATTCAAAAATAGGTAGCATACAATTAAAA
CATGGCGGGCACGTATCATTGCCCTTATCTTGTGCAGTTAGACGCGAATTTTTCGAAGAAGTACCTT
CAAAGAATGGGGTCTCATCTTGTTTTGCAAGTACCACTGAGCAGGATAATAATAGAAATGATAATATA
CTATAGTAGAGATAACGTCGATGACTTCCCATACTGTAATTGCTTTTAGTTGTGTATTTTTAGTGTGCA
AGTTTCTGTAAATCGATTAATTTTTTTTTCTTTCCTCTTTTTATTAACCTTAATTTTTATTTTAGATTCCTG
ACTTCAACTCAAGACGCACAGATATTATAACATCTGCACAATAGGCATTTGCAAGAATTACTCGTGAG
TAAGGAAAGAGTGAGGAACTATCGCATACCTGCATTTAAAGATGCCGATTTGGGCGCGAATCCTTTA
TTTTGGCTTCACCCTCATACTATTATCAGGGCCAGAAAAAGGAAGTGTTTCCCTCCTTCTTGAATTGA
TGTTACCCTCATAAAGCACGTGGCCTCTTATCGAGAAAGAAATTACCGTCGCTCGTGATTTGTTTGCA
AAAAGAACAAAACTGAAAAAACCCAGACACGCTCGACTTCCTGTCTTCCTATTGATTGCAGCTTCCAA
TTTCGTCACACAACAAGGTCCTAGCGACGGCTCACAGGTTTTGTAACAAGCAATCGAAGGTTCTGGA
ATGGCGGGAAAGGGTTTAGTACCACATGCTATGATGCCCACTGTGATCTCCAGAGCAAAGTTCGTTC
GATCGTACTGTTACTCTCTCTCTTTCAAACAGAATTGTCCGAATCGTGTGACAACAACAGCCTGTTCT
CACACACTCTTTTCTTCTAACCAAGGGGGTGGTTTAGTTTAGTAGAACCTCGTGAAACTTACATTTAC
ATATATATAAACTTGCATAAATTGGTCAATGCAAGAAATACATATTTGGTCTTTTCTAATTCGTAGTTTT
TCAAGTTCTTAGATGCTTTCTTTTTCTCTTTTTTACAGATCATCAAGGAAGTAATTATCTACTTTTTACA
ACAAATATAAAACAAGATCGGAATTCTAGAAATGTCTTGGTCTCACCCACAATTCGAAAAGGGGCCC
GGTACTAGTAGTTTGAGCGAAAGAATGCTCAAGGCTTTAAATGACCAGCTAAACAGGGAGCTTTATT
CTGCATATCTATACTTTGCCATGGCTGCCTACTTTGAAGATCTTGGCCTTGAAGGTTTCGCCAACTGG
ATGAAGGCTCAGGCTGAAGAAGAGATTGGGCATGCACTGAGGTTCTACAACTACATCTACGATCGCA
ATGGTAGGGTTGAGCTTGATGAAATTCCAAAGCCTCCAAAGGAGTGGGAGAGCCCATTAAAAGCTTT
TGAAGCTGCTTACGAGCATGAGAAATTCATAAGCAAGTCCATATATGAATTGGCAGCTTTAGCAGAG
GAGGAAAAAGATTACTCGACGAGGGCATTCTTAGAGTGGTTTATCAACGAGCAGGTTGAGGAAGAG
GCCAGCGTAAAGAAAATACTGGACAAGTTAAAGTTTGCTAAGGACAGTCCTCAAATATTGTTCATGCT
TGATAAGGAGTTGAGTGCGAGAGCTCCAAAGCTCCCAGGGCTCTTAATGCAGGGAGGAGAGTAACT
CGAGGGATCTGCGATAGATCAATTTTTTTCTTTTCTCTTTCCCCATCCTTTACGCTAAAATAATAGTTT
ATTTTATTTTTTGAATATTTTTTATTTATATACGTATATATAGACTATTATTTATCTTTTAATGATTATTAA
GATTTTTATTAAAAAAAAAATTCGCTCCTCTTTTAATGCCTTTATGCAGTTTTTTTTTCCCATTCGATATTT
CTATGTTCGGGTTCAGCGTATTTTAAGTTTAATAACTCGAAAATTCTGCGTTCGTTAAAGCTTGCATGC
CTGCAGGTCGACTCTAGAGGATCCCCGGGTACCGAGCTCGAATATTCACTGGCCGTCGTTTTACAAC
GTCGTGACTGGGAAAACCCTGGCGTTACCCAACTTAATCGCCTTGCAGCACATCCCCCTTTCGCCAG
CTGGCGTAATAGCGAAGAGGCCCGCACCGATCGCCCTTCCCAACAGTTGCGCAGCCTGAATGGCGA
ATGGCGCCTGATGCGGTATTTTCTCCTTACGCATCTGTGCGGTATTTCACACCGCATATATCGGATC
GTACTTGTTACCCATCATTGAATTTTGAACATCCGAACCTGGGAGTTTTCCCTGAAACAGATAGTATA
TTTGAACCTGTATAATAATATATAGTCTAGCGCTTTACGGAAGACAATGTATGTATTTCGGTTCCTGGA
GAAACTATTGCATCTATTGCATAGGTAATCTTGCACGTCGCATCCCCGGTTCATTTTCTGCGTTTCCA
TCTTGCACTTCAATAGCATATCTTTGTTAACGAAGCATCTGTGCTTCATTTTGTAGAACAAAAATGCAA
CGCGAGAGCGCTAATTTTTCAAACAAAGAATCTGAGCTGCATTTTTACAGAACAGAAATGCAACGCG
AAAGCGCTATTTTACCAACGAAGAATCTGTGCTTCATTTTTGTAAAACAAAAATGCAACGCGAGAGCG
CTAATTTTTCAAACAAAGAATCTGAGCTGCATTTTTACAGAACAGAAATGCAACGCGAGAGCGCTATT
TTACCAACAAAGAATCTATACTTCTTTTTTGTTCTACAAAAATGCATCCCGAGAGCGCTATTTTTCTAA
CAAAGCATCTTAGATTACTTTTTTTCTCCTTTGTGCGCTCTATAATGCAGTCTCTTGATAACTTTTTGCA
CTGTAGGTCCGTTAAGGTTAGAAGAAGGCTACTTTGGTGTCTATTTTCTCTTCCATAAAAAAAGCCTG
ACTCCACTTCCCGCGTTTACTGATTACTAGCGAAGCTGCGGGTGCATTTTTTCAAGATAAAGGCATCC
CCGATTATATTCTATACCGATGTGGATTGCGCATACTTTGTGAACAGAAAGTGATAGCGTTGATGATT

CTTCATTGGTCAGAAAATTATGAACGGTTTCTTCTATTTTGTCTCTATATACTACGTATAGGAAATGTTT
ACATTTTCGTATTGTTTTCGATTCACTCTATGAATAGTTCTTACTACAATTTTTTTGTCTAAAGAGTAAT
ACTAGAGATAAACATAAAAAATGTAGAGGTCGAGTTTAGATGCAAGTTCAAGGAGCGAAAGGTGGAT
GGGTAGGTTATATAGGGATATAGCACAGAGATATATAGCAAAGAGATACTTTTGAGCAATGTTTGTGG
AAGCGGTATTCGCAATATTTTAGTAGCTCGTTACAGTCCGGTGCGTTTTTGGTTTTTTGAAAGTGCGT
CTTCAGAGCGCTTTTGGTTTTCAAAAGCGCTCTGAAGTTCCTATACTTTCTAGCTAGAGAATAGGAAC
TTCGGAATAGGAACTTCAAAGCGTTTCCGAAAACGAGCGCTTCCGAAAATGCAACGCGAGCTGCGC
ACATACAGCTCACTGTTCACGTCGCACCTATATCTGCGTGTTGCCTGTATATATATATACATGAGAAG
AACGGCATAGTGCGTGTTTATGCTTAAATGCGTACTTATATGCGTCTATTTATGTAGGATGAAAGGTA
GTCTAGTACCTCCTGTGATATTATCCCATTCCATGCGGGGTATCGTATGCTTCCTTCAGCACTACCCT
TTAGCTGTTCTATATGCTGCCACTCCTCAATTGGATTAGTCTCATCCTTCAATGCTATCATTTCCTTTG
ATATTGGATCGATCCGATGATAAGCTGTCAAACATGAGAATTAATTCTACCCTATGAACATATTCCATT
TTGTAATTTCGTGTCGTTTCTATTATGAATTTCATTTATAAAGTTTATGTACACGTACGCTGCAGGTCG
ACCGTACGCTGCAGGTCGACGGATCCCCGGGTTAATTAAGGCGCGCCAGATCTGTTTAGCTTGCCT
CGTCCCCGCCGGGTCACCCGGCCAGCGACATGGAGGCCCAGAATACCCTCCTTGACAGTCTTGAC
GTGCGCAGCTCAGGGGCATGATGTGACTGTCGCCCGTACATTTAGCCCATACATCCCCATGTATAAT
CATTTGCATCCATACATTTTGATGGCCGCACGGCGCGAAGCAAAAATTACGGCTCCTCGCTGCAGAC
CTGCGAGCAGGGAAACGCTCCCCTCACAGACGCGTTGAATTGTCCCCACGCCGCGCCCCTGTAGA
GAAATATAAAAGGTTAGGATTTGCCACTGAGGTTCTTCTTTCATATACTTCCTTTTAAAATCTTGCTAG
GATACAGTTCTCACATCACATCCGAACATAAACAACCATGGGTAAGGAAAAGACTCACGTTTCGAGG
CCGCGATTAAATTCCAACATGGATGCTGATTTATATGGGTATAAATGGGCTCGCGATAATGTCGGGC
AATCAGGTGCGACAATCTATCGATTGTATGGGAAGCCCGATGCGCCAGAGTTGTTTCTGAAACATGG
CAAAGGTAGCGTTGCCAATGATGTTACAGATGAGATGGTCAGACTAAACTGGCTGACGGAATTTATG
CCTCTTCCGACCATCAAGCATTTTATCCGTACTCCTGATGATGCATGGTTACTCACCACTGCGATCCC
CGGCAAAACAGCATTCCAGGTATTAGAAGAATATCCTGATTCAGGTGAAAATATTGTTGATGCGCTG
GCAGTGTTCCTGCGCCGGTTGCATTCGATTCCTGTTTGTAATTGTCCTTTTAACAGCGATCGCGTATT
TCGTCTCGCTCAGGCGCAATCACGAATGAATAACGGTTTGGTTGATGCGAGTGATTTTGATGACGAG
CGTAATGGCTGGCCTGTTGAACAAGTCTGGAAAGAAATGCATAAGCTTTTGCCATTCTCACCGGATT
CAGTCGTCACTCATGGTGATTTCTCACTTGATAACCTTATTTTTGACGAGGGGAAATTAATAGGTTGT
ATTGATGTTGGACGAGTCGGAATCGCAGACCGATACCAGGATCTTGCCATCCTATGGAACTGCCTCG
GTGAGTTTTCTCCTTCATTACAGAAACGGCTTTTTCAAAAATATGGTATTGATAATCCTGATATGAATA
AATTGCAGTTTCATTTGATGCTCGATGAGTTTTTCTAATCAGTACTGACAATAAAAAGATTCTTGTTTT
CAAGAACTTGTCATTTGTATAGTTTTTTTATATTGTAGTTGTTCTATTTTAATCAAATGTTAGCGTGATT
TATATTTTTTTTCGCCTCGACATCATCTGCCCAGATGCGAAGTTAAGTGCGCAGAAAGTAATATCATG
CGTCAATCGTATGTGAATGCTGGTCGCTATACTGCTGTCGATTCGATACTAACGCCGCCATCCAGTG
TCGAAAACGAGCTCGATTCATCGATGACGTCAGGTGGCACTTTTCGGGGAAATGTGCGCGGAACCC
CTATTTGTTTATTTTTCTAAATACATTCAAATATGTATCCGCTCATGAGACAATAACCCTGATAAATGCT
TCAATAATATTGAAAAAGGAAGAGTATGAGTATTCAACATTTCCGTGTCGCCCTTATTCCCTTTTTTGC
GGCATTTTGCCTTCCTGTTTTTGCTCACCCAGAAACGCTGGTGAAAGTAAAAGATGCTGAAGATCAGT
TGGGTGCACGAGTGGGTTACATCGAACTGGATCTCAACAGCGGTAAGATCCTTGAGAGTTTTCGCCC
CGAAGAACGTTTTCCAATGATGAGCACTTTTAAAGTTCTGCTATGTGGCGCGGTATTATCCCGTATTG
ACGCCGGGCAAGAGCAACTCGGTCGCCGCATACACTATTCTCAGAATGACTTGGTTGAGTACTCACC
AGTCACAGAAAAGCATCTTACGGATGGCATGACAGTAAGAGAATTATGCAGTGCTGCCATAACCATG
AGTGATAACACTGCGGCCAACTTACTTCTGACAACGATCGGAGGACCGAAGGAGCTAACCGCTTTTT
TGCACAACATGGGGGATCATGTAACTCGCCTTGATCGTTGGGAACCGGAGCTGAATGAAGCCATAC
CAAACGACGAGCGTGACACCACGATGCCTGTAGCAATGGCAACAACGTTGCGCAAACTATTAACTG
GCGAACTACTTACTCTAGCTTCCCGGCAACAATTAATAGACTGGATGGAGGCGGATAAAGTTGCAGG
ACCACTTCTGCGCTCGGCCCTTCCGGCTGGCTGGTTTATTGCTGATAAATCTGGAGCCGGTGAGCG
TGGGTCTCGCGGTATCATTGCAGCACTGGGGCCAGATGGTAAGCCCTCCCGTATCGTAGTTATCTAC
ACGACGGGGAGTCAGGCAACTATGGATGAACGAAATAGACAGATCGCTGAGATAGGTGCCTCACTG
ATTAAGCATTGGTAACTGTCAGACCAAGTTTACTCATATATACTTTAGATTGATTTAAAACTTCATTTTT
AATTTAAAAGGATCTAGGTGAAGATCCTTTTTGATAATCTCATGACCAAAATCCCTTAACGTGAGTTTT
CGTTCCACTGAGCGTCAGACCCCGTAGAAAAGATCAAAGGATCTTCTTGAGATCCTTTTTTTCTGCGC
GTAATCTGCTGCTTGCAAACAAAAAAACCACCGCTACCAGCGGTGGTTTGTTTGCCGGATCAAGAGC
TACCAACTCTTTTTCCGAAGGTAACTGGCTTCAGCAGAGCGCAGATACCAAATACTGTCCTTCTAGTG
TAGCCGTAGTTAGGCCACCACTTCAAGAACTCTGTAGCACCGCCTACATACCTCGCTCTGCTAATCC

TGTTACCAGTGGCTGCTGCCAGTGGCGATAAGTCGTGTCTTACCGGGTTGGACTCAAGACGATAGTT
ACCGGATAAGGCGCAGCGGTCGGGCTGAACGGGGGGTTCGTGCACACAGCCCAGCTTGGAGCGAA
CGACCTACACCGAACTGAGATACCTACAGCGTGAGCTATGAGAAAGCGCCACGCTTCCCGAAGGGA
GAAAGGCGGACAGGTATCCGGTAAGCGGCAGGGTCGGAACAGGAGAGCGCACGAGGGAGCTTCCA
GGGGGAAACGCCTGGTATCTTTATAGTCCTGTCGGGTTTCGCCACCTCTGACTTGAGCGTCGATTTT
TGTGATGCTCGTCAGGGGGGCGGAGCCTATGGAAAAACGCCAGCAACGCGGCCTTTTTACGGTTCC
TGGCCTTTTGCTGGCCTTTTGCTCACATGTTCTTTCCTGCGTTATCCCCTGATTCTGTGGATAACCGT
ATTACCGCCTTTGAGTGAGCTGATACCGCTCGCCGCAGCCGAACGACCGAGCGCAGCGAGTCAGTG
AGCGAGGAAGCGGAAGA

Supplementary Table 4. DNA sequence of SA optimized for SPFt-based biosensing

ATGGCTAGCATGACTGGTGGACAGCAAATGGGTCGCGACCAGGAGGCCGGCATCACCGGCACCTG
GTACAACCAGCTCGGCTCGACCTTCATCGTGACCGCGGGCGCCGACGGCGCCCTGACCGGAACCT
ACGTGACCGCTCGCGGCAACGCCGAGAGCCGCTACGTCCTGACCGGTCGTTACGACAGCGCCCCG
GCCACCGACGGCAGCGGCACCGCCCTCGGTTGGACGGTGGCCTGGAAGAATAACTACCGCAACGC
CCACTCCGCGACCACGTGGAGCGGCCAGTACGTCGGCGGCGCCGAGGCGAGGATCAACACCCAGT
GGCTGCTGACCTCCGGCACCACCGAGGCCAACGCCGCGAAGTCCACGCTGGTCGGCCACGACACC
TTCACCAAGGTGAAGCCGTCCGCCGCCTCCATCGACGCGGCGAAGAAGGCCGGCGTCAACAACGG
CAACCCGCTCGACGCCGTTCAGCAGTAA
